# Risk factors for severe adverse drug reactions in hospitalized patients

**DOI:** 10.1515/med-2024-1122

**Published:** 2025-01-13

**Authors:** Nemanja Z. Petrović, Ana V. Pejčić, Miloš N. Milosavljević, Slobodan M. Janković

**Affiliations:** Department of Pharmacology and Toxicology, Faculty of Medical Sciences, University of Kragujevac, Svetozara Markovića 69, 34000, Kragujevac, Serbia; Department of Clinical Pharmacology, University Clinical Center Kragujevac, 34000, Kragujevac, Serbia

**Keywords:** severe adverse drug reactions, risk factors, drugs, polypharmacy

## Abstract

**Background:**

Severe adverse drug reactions (sADRs) are becoming increasingly common nowadays. The incidence of sADRs is approaching 6.7%, and the incidence of fatal adverse reactions is 0.32% in hospitalized patients. Of these, 48.5% are, at least potentially, preventable.

**Aims:**

This study’s objective was to determine factors associated with the occurrence and preventability of sADRs occurring at the tertiary level.

**Methods:**

A case-control retrospective-prospective clinical observational study design was used for the study. The research cohort included patients hospitalized at the University Clinical Center (UCC) in Kragujevac, Serbia, from January 1, 2019 to January 1, 2024. The research comprised 147 individuals who were admitted to the UCC in Kragujevac. There were 49 patients with sADRs and 98 controls.

**Results:**

Significant factors associated with sADRs in our study were a total number of consultations (ORadjusted = 5.60), Charlson comorbidity index (ORadjusted = 0.30), C-reactive protein (ORadjusted = 1.07), prescribed antihistamines (ORadjusted = 14.37), and antihypertensives (ORadjusted = 0.15).

**Conclusion:**

We have identified the factors that are associated with sADRs should be kept in mind while working with patients at the tertiary level. Early detection of those factors may help with early notification of sADRs and their prevention.

## 1 Introduction

Severe adverse drug reactions (ADRs) are adverse reactions associated with the use of a drug when the outcome of the patient includes circumstances such as death, development of life-threatening diseases, hospitalization or its prolongation, disability or permanent damage, congenital anomaly of the offspring of the treated patient, or the need for interventional measures aimed at preventing the consequences [1].

Because of their potential to significantly increase morbidity, mortality, and economic burden, sADRs are significant public health problem [2]. Adverse drug reactions are the fourth among the six leading causes of death worldwide, taking their place among such causes as heart disease, cancer, and stroke [3]. The incidence of severe adverse reactions is 6.7%, and the incidence of fatal adverse reactions is 0.32% in hospitalized patients [4]. In one study, sADRs were shown to account for 3.6% of hospital admissions. Of these, 48.5% were, at least potentially, preventable. For more than half of the patients who experienced severe side effects, admission to the hospital or a visit to the emergency room was indicated. The most severe adverse reactions reported involved gastrointestinal system [5].

The known risk factors for sADRs are polypharmacy, impaired liver function, age of the patient, alcohol consumption, sex, race, pregnancy, breastfeeding, kidney injury, dose, and frequency of drug administration [6,7,8,9].

Understanding severe drug side effects is critical to patient safety and effective healthcare management [10]. It helps monitor the safety of drugs and take necessary actions to mitigate the risks associated with their use [11].

Only a few studies were done on this topic at the tertiary care level and limited number of factors was found to contribute to emergence of sADRs; besides, certain risk factors remained un-investigated or unquantified precisely.

This study aims to determine factors associated with the occurrence and preventability of sADRs in the hospital setting.

## 2 Methods

A retrospective-prospective clinical observational study with case–control study design was used for the study. The patients included in the study were those who had been admitted to the University Clinical Center (UCC) in Kragujevac, Serbia, from January 1, 2019 to January 1, 2024. The cases were patients experiencing sADRs estimated at least as “probable” by the Naranjo causality assessment scale [12]. Controls were patients without sADRs. Controls were matched with cases in a ratio of 1:2 The conditions needed for patients to be included were: hospitalized at UCC Kragujevac, having prescribed medication, and complete patient files. Patients who were under the age of 18, whose medical history had incomplete information, or who had overdosed were not allowed to participate in the research. The study’s sample was convenient in that it was consecutive; all patients who met the inclusion criteria and were not excluded throughout the study period were included.

The primary research outcome was emergence of a sADR. Preventability was assessed using the Schumock and Thornton scale [13]. Aside from a number of independent factors identified by earlier studies as potentially influencing sADRs (liver function impairment, age of the patient, sex, andkidney injury), numerous other confounders were also taken out of the patient data, including regular hematology and blood biochemistry, the Charlson comorbidity index, and prescription medications, number of consulted doctors, total number of consultations, previous occurrence of an adverse effect, length of hospitalization, recent surgery, and whether the patient was in intensive care. Two separate researchers retrieved the data from the computerized patient histories stored in the hospital information system “ZIS” (Comtrade, Belgrade), and by consensus, they were harmonized with each other. A small sample size enough to determine the variables that are substantially linked to a sADRs, which is the primary result determined by utilizing the G Power 3.1.9.7 program [14] based on the result of a previously conducted study [15]. The computation was performed using the following inputs: probability of type 1 error of 0.05, minimal statistical power 0.8, odds ratio: 6.87, frequency of outcome: 0.221, R2: 0.4, and distribution: normal. Based on all these parameters, the required minimum calculated size of the case groups was 15 patients, while the control should be twice as many. The information gathered from the ZIS system was first separately coded numerically, tabulated, and error-checked by the two investigators. Next, measures of central tendency and variability (if continuous) or frequencies, relative numbers, and percentages (if categorical) were used to describe the data. While the median and interquartile range depicted the data dispersed in another way, the mean and standard deviation were employed to explain continuously distributed data that was normally distributed. Multivariate binary logistic regression was used to examine the impact of potential predictors and confounders on the study’s results. The satisfaction of the following presumptions was verified prior to the application of these multivariate techniques: binary outcome, independence of observations, lack of multicollinearity, absence of severe outliers, and a sizable enough sample size for multivariate binary logistic regression). Cox & Snell *R* square, Nagelkerke *R* square, and Hosmer and Lemeshow test were used to evaluate the regression models’ quality. If the probability of the null hypothesis was ≤0.05, the results were deemed statistically significant. The Statistical Package for the Social Sciences, version 18.0, was used for all computations.


**Ethical approval:** The study was performed in line with all the relevant national regulations, institutional policies, and in accordance with the tenets of the Helsinki Declaration and has been reviewed and approved by the institutional review board, the Ethics Committee of University Clinical Center Kragujevac *[approval number: 01/24-57].*

**Informed consent**: Written informed consent was obtained from all individual participants included in the study.

## 3 Results

A total of 147 hospitalized patients receiving care at the UCC in Kragujevac were included in the research. The number of cases that had sADR was 49 (33.3%), and the total number of controls was 98 (66.7%). Three patients (2%) died. Life-threatening severe adverse reaction was experienced by 21 patients (14.3%). Twenty-five patients required prolonged hospitalization (17%). The data on thecharacteristics of the study sample, recorded sADRs and their preventability are shown in Tables 1–3. Table 1 shows demographic and clinical data for the whole study sample. The identified sADRs presented in Table 2 are then classified according to their preventability in Table 3 (definitely preventable, probably preventable, and not preventable).

**Table 1:** Characteristics of respondents

Variable	Frequency (percentage) for categorical variables, Mean ± SD, median, IQR for continuous variables
Gender (m/f)	74/73 ( 50.3%/49.7%)
Age	62.50 ± 13.180, 63, 14
sADRs (yes/no)	49/98 (33.3%/66.7%)
Death (yes/no)	3/144 (2%/98%)
Life-threatening (yes/no)	21/126 (14.3%/85.7%)
Requires or prolong hospitalization (yes/no)	25/122 (17%/83%)
Length of hospitalization	10.84 ± 3.52, 10, 5
Number of consulted doctors	4.18 ± 1.69, 4, 2
Total number of consultations	6.06 ± 2.49, 6, 3
Previous occurrence of an adverse effect (yes/no)	14/133 (9.5%/90.5%)
Intensive care (yes/no)	42/105 (28.6%/71.4%)
Diabetes mellitus 2 (yes/no)	70/77 (47.6%/52.4%)
Myocardial infarction (yes/no)	39/108 (26.5%/73.5%)
Congestive heart failure (yes/no)	73/74 (49.7%/50.3%)
Peripheral vascular disease (yes/no)	51/96 (34.7%/65.3)
Cerebrovascular event (yes/no)	23/124 (15.6%/84.4%)
Dementia (yes/no)	13/134 (8.8%/91.2%)
Chronic obstructive pulmonary disease (yes/no)	32/115 (21.8%/78.2%)
Recent abdominal surgeries (yes/no)	11/136 (7.5%/92.5%)
Gastric ulcer (yes/no)	31/116 (21.1%/78.9%)
Liver disease (yes/no)	15/132 (10.2%/89.8%)
Hemiplegia (yes/no)	4/143 (2.7%/97.3%)
Solid tumor (yes/no)	28/119 (19%/81%)
Chronic kidney injury (yes/no)	26/121 (17.7%/82.3%)
Antihypertensives (yes/no)	83/64 (56.5%/43.5%)
Antiepileptics (yes/no)	37/110 (25.2%/74.8%)
NSAIDs (yes/no)	86/61 (58.5%/41.5%)
Corticosteroids (yes/no)	52/95 (35.4%/64.6%)
Antihistamines (yes/no)	57/90 (38.8%/61.2%)
Platelets	248.44 ± 65.23, 246, 96
Erythrocytes	3.77 ± 0.85, 3.41, 1.17
Serum creatinine	84.84 ± 38.82, 75, 40
Sodium	140.02 ± 7.46, 139, 7
Potassium	4.101 ± 0.73, 4.1, 0.8
Hematocrit	0.33 ± 0.06, 0.33, 0.08
Leukocytes	9.21 ± 3.57, 9.1, 4.8
Hemoglobin	105.21 ± 22.6, 98, 36
ALT	27.38 ± 28.4, 21, 17
AST	29.81 ± 31.4, 24, 17
Glycemia	8.4 ± 4.55, 7.9, 3.2
CRP	15.14 ± 28.76, 8, 7
Charlson comorbidity index	6.75 ± 1.91, 7, 2

Abbreviations: NSAIDs – non-steroidal anti-inflammatory drugs; AST – aspartate aminotransferase; ALT – alanine aminotransferase; CRP – C-reactive protein. Note: Results for continuous variables are shown as mean ± standard deviation (SD), median, interquartile range (IQR), and for categorical variables as frequency and percentages.

**Table 2:** The most common sADRs

Parameter	Frequency	Drug
Stevens–Johnson syndrome	1/49	Co-trimoxazole
Iatrogenic Cushing’s syndrome	9/49	Glucocorticoids
Drug-induced anaphylaxis	8/49	Ibuprofen
Drug-induced thyroiditis/thyroid storm	7/49	Amiodarone
Drug-induced nonautoimmune hemolytic anemia	3/49	Methyldopa
Drug-induced cataract	5/49	Glucocorticoid
Generalized skin eruption due to drugs and medicaments taken internally	4/49	Ibuprofen
Drug-induced systemic lupus erythematosus	4/49	Quinidine
Drug-induced osteoporosis with pathological fracture	8/49	Glucocorticoids

**Table 3:** Preventability of sADRs

Category	No. of serious ADRs	Percentage (%)
**Definitely preventable**	10	20.4
Stevens–Johnson syndrome	1	2
Drug-induced cataract	5	10.2
Drug-induced systemic lupus erythematosus	4	8.2
**Probably preventable**	21	42.9
Iatrogenic Cushing’s syndrome	9	18.4
Drug-induced anaphylaxis	4	8.2
Drug-induced osteoporosis with pathological fracture	8	16.3
**Not preventable**	18	36.7
Drug-induced anaphylaxis	4	8.2
Drug-induced thyroiditis/thyroid storm	7	14.2
Generalized skin eruption due to drugs and medicaments taken internally	4	8.2
Drug-induced nonautoimmune hemolytic anemia	3	6.1

The relationship between independent and confounding factors and sADRs was investigated by multivariate binary logistic regression. Using a forward conditional stepwise method, the model was constructed with the whole set of possible predictors: number of consulted physicians, total number of consultations, Charlson comorbidity index, alanine aminotransferase (ALT), C-reactive protein (CRP), prescribed antihistamines, antihypertensives, opioids, non-steroidal anti-inflammatory drugs (NSAIDs), hemoglobin level, procalcitonin level, andgender.

The following assumptions for logistic regression were met: no extreme outliers, a sufficiently large sample, no multicollinearity (variance inflation factor (VIF) < 2 for all predictors), independency of observations, binary outcome (sADRs or not), and no extreme outliers. The Box–Tidwell test verified for each variable the linear connection between the explanatory factors and the logit of the results. The factors listed in Table 4 were included in the final binary logistic regression model, which provided a good fit for the data.: Hosmer and Lemeshow test was 1.729 (df = 8, *p* = 0.988), Cox & Snell *R* square 0.628, and Nagelkerke *R* square 0.872. The Table 4 shows strength of association between predictors and the severe ADRs: the crude odds ratio reflects isolated association, while the adjusted odds ratio (OR adjusted) reflects the association adjusted for simultaneous influence of other predictors. Factors that increase likelihood of severe ADRs are total number of consultations (OR adujsted 5.598), CRP (OR adjusted 1.070) and administration of antihistamines (OR adjusted 14.368), while Charlson comorbidity index (OR adjusted 0.298) and administration of antihypertensives (OR adjusted 0.149) exerted protective effects.

**Table 4:** Multivariate analysis according to outcomes

Parameter	OR crude	OR adjusted	*p*
**sADRs**			
Total number of consultations	4.220 (2.560–6.957)	5.598 (2.335–13.420)	0.00
Charlson comorbidity index	0.680 (0.554–0.835)	0.298 (0.143–0.621)	0.01
CRP	1.056 (1.007–1.106)	1.070 (1.001–1.144)	0.04
Antihistamines	10.154 (4.557–22.527)	14.368 (2.580–80.007)	0.02
Antihypertensives	0.336 (0.166–0.684)	0.149 (0.028–0.807)	0.03

## 4 Discussion

Significant factors associated with sADRs in our study were a total number of consultations, Charlson comorbidity index, CRP, prescribed antihistamines, and antihypertensives. Previous studies have found that increased number of consultations in the primary care setting is very common in patients with adverse drug reactions [16]. More consultations often mean more medications being prescribed, which increases the likelihood of ADRs. Frequent consultations can lead to polypharmacy (the use of multiple medications by a patient), which is a known risk factor for ADRs. The more medications a patient is prescribed, the higher the chance of drug–drug interactions, which can result in ADRs [17,18]. Relatively small number of patients with severe ADRs identified in our study (compared to average annual admission rate of 50,000) reflects their underreporting (the systematic review by Hazell and Shakir reported that 95% of severe ADRs among hospitalized patients are not reported), and incompleteness of patients’ files [19].

A significant risk factor for the origin of sADRs in our study was CRP. High CRP levels may indicate an overactive immune response, which can exacerbate drug reactions. Elevated CRP can be a predictive marker for complications, including severe drug reactions, especially in patients with numerous comorbidities. CRP levels increase in response to inflammation, which can trigger an adverse reaction to drugs by the mechanisms that affect the liver and other organs [20,21,22].

A prescribed antihistamine is another risk factor associated with the appearance of sADRs. These drugs may also have anticholinergic effects, which can cause side effects like dry mouth, urinary retention, and in severe cases, toxicity [23,24,25].

It has been found that the Charlson comorbidity index protects from sADRs. Since our study was carried out at the tertiary level, it is probable that doctors paid greater attention to patients who had more comorbidities and more frequently gave these patients the proper prophylactic for sADRs.

Prescribed antihypertensives also protect from sADRs in our study. Propranolol, a non-cardioselective β-blocker, is the usual treatment for thyrotoxic crisis and simple hyperthyroidism [26]. A high dose of propranolol can gradually lower serum triiodothyronine (T3) concentrations by inhibiting the conversion of T4 to T3 [27]. Perhaps its protective effect on heart may also mitigate adverse drug reactions involving this organ.

Our study also showed that almost two-thirds of all severe ADRs were either definitely or probably preventable, which sends a message to clinicians that majority of severe ADRs could be prevented if they are thought of when prescribing drugs to inpatients. Routine check-up of sections “Contraindications” and “Warnings” of summaries of product characteristics when prescribing to inpatients could help reminding ourselves to possible severe ADRs; if this is followed by investigation of risk factors identified in this study, the chances of avoiding severe ADRs are significantly increased.

This study has several limitations. Due to the influence of local circumstances on results (e.g., local treatment protocols, unequal practices of doctors, unavailability of particular medications, etc.), this unicentric study is susceptible to bias. This study’s limited sample size and small statistical strength have made it possible for results to be erroneously negative, as in the case of sunitinib, which was prescribed to a small number of patients.

## 5 Conclusions

The factors have shown to be related with sADRs should be considered while working with patients at the tertiary care level. Early detection of these factors may help with early notification of sADRs and their prevention.
